# Analytical Assessment of the Propagation of Colored Sensor Noise in Strapdown Inertial Navigation

**DOI:** 10.3390/s20236914

**Published:** 2020-12-03

**Authors:** Christopher Blum, Johann Dambeck

**Affiliations:** Department of Aerospace and Geodesy, Institute of Flight System Dynamics, Technical University of Munich, 85748 Garching, Germany; dambeck@tum.de

**Keywords:** inertial navigation, inertial measurement unit, sensor errors, navigation performance, noise processes, error propagation, angular random walk, bias instability

## Abstract

Knowledge of the propagation of sensor errors in strapdown inertial navigation is crucial for the design of inertial and integrated navigation systems. The propagation of initialization errors and deterministic sensor errors is well covered in the literature. If considered at all, the propagation of inertial sensor noise has typically been assessed for un-correlated (white) Gaussian noise. Real inertial sensor noise, however, is time-correlated (colored) and best described by a combination of different stochastic processes. In this paper, we demonstrate how a navigation system’s response to colored noise input differs from the response to bias-like or white noise inputs. We present a method for assessing the navigation error from various inertial sensor noise processes without the need for time-consuming Monte Carlo simulations and demonstrate its application and validity with real sensor data. The proposed method is used to determine in which scenarios the sensor’s real noise can be approximated by simple white Gaussian noise. The results indicate that neglecting colored sensor noise is justified for many applications, but should be checked individually for each sensor configuration and mission.

## 1. Introduction

Selecting suitable inertial sensors for an inertial or integrated navigation system is a crucial step in the system’s design. Clearly, this step requires in-depth understanding of the propagation of inertial measurement errors within the navigation algorithms. Typically, a general sensor error model of the following or similar structure is used to describe the specific forces $\tilde{f}{}_{b}$ and angular rate $\tilde{\omega }{}_{ib}$ measurements from the true states $f{}_{b},\;\omega {}_{ib}$, in the simulation and analysis of navigation systems:

(1) $$\begin{array}{cc} \tilde{f}{}_{b} & =b{}_{a}+M{}_{a}f{}_{b}+\nu {}_{a} \end{array}$$

(2) $$\begin{array}{cc} \tilde{\omega }{}_{ib} & =b{}_{g}+M{}_{g}\omega {}_{ib}+\nu {}_{g} \end{array}$$

These models include sensor biases $b{}_{}$, a scale-factor and misalignment matrix $M{}_{}$ and noise terms $\nu {}_{}$ for each accelerometer and gyroscope axis. Depending on the application, these simple models are extended by higher-order errors terms and environmental influences. In many cases, the noise terms are simply approximated as white Gaussian noise [1].

The growth of navigation state errors (position, velocity and orientation) from the above described inertial sensor errors is defined by the navigation system’s error dynamics. The error dynamics of platform and, more importantly, strapdown inertial navigation systems is, in general, well covered in the literature. An extensive discussion of inertial navigation error dynamics is, e.g., given in the works of Britting [2], Savage [3] and Chatfield [4]. This includes analytical expressions of the position error’s growth from both initialization errors and sensor biases. Short- and medium-term approximations of these expressions can also be found in [1,5,6]. While these allow for the analysis of bias-like errors e.g., run-to-run bias variations, the system’s response to noise-like errors is rarely covered. With the advent of optical gyroscopes, the random walk noise became more significant compared to the previous mechanical gyroscopes, which leads to increasing interest in the propagation of gyro noise [7]. With the proliferation of micro-electro-mechanical system (MEMS) sensors and their complex error behavior [8,9], considering colored noise and especially long-term processes has again gained in importance. Still, publications are limited to considering white Gaussian noise, e.g., [3,7] or quantization noise of integrating sensors [10] for predicting the navigation performance.

In reality, however, the measurement noise of inertial sensors indeed contains time-correlated components that are represented by various noise processes, as pointed out in e.g., [11,12,13]. State of the art is the identification and analysis of the sensor noise processes using the power spectral density (PSD) and Allan variance [14] as demonstrated in [15,16,17]. Based on these two methods, the Institute of Electrical and Electronics Engineers (IEEE) standards on specification and testing of various inertial sensor technologies [18,19,20,21] define five typical noise processes that can be found in inertial sensor noise and is covered in this publication:

- angular random walk,
- rate random walk,
- in-run bias instability,
- rate ramp noise,
- quantization noise.

Despite the existence of methods that consider these sensor noise processes in a Kalman filter framework [12,22] to increase estimation consistency, the actual influence of colored sensor noise on the inertial position drift (e.g., between two updates) is not well covered. The often-utilized white noise model represents only one of the different processes, namely the angular random walk for gyroscopes, respectively, velocity random walk for accelerometers. This obvious discrepancy between the typical modeling and real sensor behavior raises two questions that shall be answered within this paper:

- How do the these sensor noise processes propagate through the strapdown inertial navigation?
- Under what circumstances is neglecting non-white noise processes actually justified?

Of course, these questions could be answered by numerical simulation. A discussion of detailed sensor noise modeling for numerical simulations can be found e.g., in [23]. Such a numerical simulation can provide highly accurate results, but requires detailed modeling, is time-consuming and provides little insight into the underlying mechanisms compared to the analytical modeling.

Within this manuscript, we present a more basic and simple-to-use method for evaluating the navigation errors from a sensor’s noise properties. The proposed method is not meant to replace the high detail Monte Carlo simulations that are used to demonstrate the navigation performance, but to allow a first assessment of the navigation errors caused by the sensor noise. For that, an analytical model of the inertial navigation system’s response to the various sensor noise processes is derived within the first section of this publication. This extends the already known analytical solutions for bias-like errors and white noise by analytical solutions for the most typical (non-white) sensor noise processes. Subsequently, the various results for sensor error propagation are presented and validated using real sensor measurements. Finally, the results are used to determine for which applications and under what conditions the various noise processes may be neglected compared to the white noise components.

## 2. Propagation of Sensor Noise in Strapdown Inertial Navigation

An inertial navigation system’s response to stochastic input, like sensor noise, is of course of stochastic nature and requires respective methods of analysis. A performance analysis and demonstration of a designed navigation system is usually performed using time-consuming Monte Carlo simulations of representative mission scenarios. This work, however, aims at providing an application-independent, more general insight into propagating inertial sensor noise processes. Therefore, we revisit the analytical representation of strapdown navigation’s error dynamics and derive the system’s white noise response and then extend these to the typical sensor noise processes.

### 2.1. Strapdown Inertial Navigation Error Dynamics

A strapdown inertial navigation algorithm propagates a vehicle’s position, velocity and orientation based on the measured specific forces (accelerations) $f{}_{b}$ and angular rates $\omega {}_{ib}$ of the vehicle’s body with respect to the inertial reference frame. For the sake of vividness and simple interpretation, this analysis is based on a strapdown inertial algorithm in (local leveled) navigation frame mechanization. This has the inherent advantage of separated horizontal and vertical channels. In the selected mechanization, propagating the position in geodetic coordinates (latitude ϕ, longitude λ, altitude *h*) is described by the following set of coupled differential equations [6]:

(3) $$\dot{\lambda }{}_{}=\left[\begin{array}{c} \dot{\phi }\\ \dot{\lambda }\\ \dot{h} \end{array}\right]=\underset{D{}_{}\left(\lambda {}_{},v{}_{n}\right))}{\underset{︸}{\left[\begin{array}{c} \frac{v_{n}}{R_{M}\left(\phi \right)+h}\\ \frac{v_{e}}{\left(R_{N}\left(\phi \right)+h\right)Cos\left(\phi \right)}\\ -v_{d} \end{array}\right]}}$$

with the north, east and down velocities $v_{n},v_{e},v_{d}$ and the local meridional and normal Earth radii $R_{M}$ and $R_{N}$. Based on the vehicle’s orientation and the measured specific forces, the change of velocity in the local North–East–Down (NED) frame is given as:

(4) $$\dot{v}{}_{n}=\left[\begin{array}{c} {\dot{v}}_{n}\\ {\dot{v}}_{e}\\ {\dot{v}}_{d} \end{array}\right]=R{}_{nb}f{}_{b}-\left(2R{}_{ne}\Omega_{ie}R{}_{en}+\Omega_{en}\right)v{}_{n}+\gamma {}_{n}\left(\phi ,\lambda ,h\right)$$

where $R{}_{nb}$ denotes the rotation matrix from the body fixed *b*-frame to the local north-east-down *n*-frame, $R{}_{ne}$ from the Earth-centered Earth-fixed (ECEF) *e*-frame to the *n*-frame. $\Omega_{ie}$ and $\Omega_{en}$ are the skew-symmetric matrices of the respective angular rate vectors $\omega {}_{ie}$ and $\omega {}_{en}$. Due to the moving reference system, the measured accelerations are corrected for Coriolis and centrifugal forces. The local gravity $\gamma {}_{n}$ is typically from gravity models like Somigliana’s gravity formula [24] or higher-order models like the EGM2008 [25], depending on the application. The vehicle’s change of orientation $R{}_{nb}$ with respect to the local North–East–Down (NED) frame is described by the following orientation differential equation:

(5) $$\dot{R}{}_{nb}=R{}_{nb}\Omega_{ib}-\Omega_{in}R{}_{nb}$$

The rotation matrix from the Earth-fixed frame *e* to the local leveled *n*-frame depends on the vehicle’s geodetic position and is given by:

(6) $$R{}_{ne}=\left[\begin{array}{ccc} -\sin\phi \cos\lambda & -\sin\phi \sin\phi & \cos\phi \\ \sin\lambda & \cos\lambda & 0\\ -\cos\phi \cos\lambda & -\cos\phi \sin\lambda & -\sin\phi \end{array}\right]$$

The corresponding transport rate in the navigation frame is given by:

(7) $$\omega {}_{en}={\left[\begin{array}{ccc} \dot{\lambda }\cos\phi & -\dot{\phi } & -\dot{\lambda }\sin\phi \end{array}\right]}^{⊺}$$

We are aware that the above strapdown inertial formulation would require modifications for a real-world implementation due to the singularities at the Earth’s poles and a computationally non-optimal orientation representation. However, this choice allows for a comprehensible and representative analysis of the dynamics of strapdown inertial navigation systems.

By splitting the true navigation states from (3) to (5) into the estimated (marked by the hat) and error states (marked by the δ), a set of differential equations for the dynamics of the error states is derived. Although, in contrast to the simple separation of the position and velocity states, the orientation error states are represented by multiplying a preceding error rotation matrix $R{}_{n\hat{n}}$:

(8) $$\begin{array}{cc} \lambda {}_{} & =\hat{\lambda }{}_{}+\delta \lambda {}_{} \end{array}$$

(9) $$\begin{array}{cc} v{}_{n} & =\hat{v}{}_{n}+\delta v{}_{n} \end{array}$$

(10) $$\begin{array}{cc} R{}_{nb} & =R{}_{n\hat{n}}R{}_{\hat{n}b} \end{array}$$

Applying above definitions to the strapdown Equations (3)–(5) and solving for the error states yields the differential equations of the strapdown error dynamics. Linearization of the error state differential equations yields the following set of linear ordinary differential equations:

(11) $$\begin{array}{cc} \delta \dot{\lambda }{}_{} & \approx D{}_{(}\phi {}_{,}{h)}^{-1}\delta v{}_{n} \end{array}$$

(12) $$\begin{array}{c} \begin{array}{cc} \delta \dot{v}{}_{n} & \approx \left(\frac{\partial \gamma {}_{}\left(\lambda {}_{}\right)}{\partial \delta \lambda {{}_{}}^{⊺}}+\frac{\partial \left(2R{}_{ne}\left(\lambda {}_{}\right)\omega {}_{ie}+\omega {}_{en}\left(\lambda {}_{},v{}_{n}\right)\right)\times v{}_{n}}{\partial \delta \lambda {{}_{}}^{⊺}}\right)\delta \lambda {}_{}\\ \; & \;+\frac{\partial \left(2R{}_{ne}\left(\lambda {}_{}\right)\omega {}_{ie}+\omega {}_{en}\left(\lambda {}_{},v{}_{n}\right)\right)\times v{}_{n}}{\partial \delta v{{}_{n}}^{⊺}}\;\delta v{}_{n}-\left(R{}_{nb}f{}_{b}\times \right)\delta \psi {}_{nn}+R{}_{nb}\delta f{}_{b}+\delta \gamma {}_{n} \end{array} \end{array}$$

(13) $$\begin{array}{cc} \dot{\psi }{}_{n\tilde{n}} & \approx -\Omega_{in}\left(\lambda {}_{},\tilde{v}{}_{n}\right)\psi {}_{n\tilde{n}}+R{}_{\tilde{n}b}\delta \omega {}_{ib}-\frac{\partial \omega {}_{in}\left(\lambda {}_{},v{}_{n}\right)}{\partial \lambda {}_{{}^{⊺}}}\delta \lambda {}_{}-\frac{\partial \omega {}_{in}\left(\lambda {}_{},v{}_{n}\right)}{\partial v{{}_{n}}^{⊺}}\delta v{}_{n} \end{array}$$

where the orientation error matrix $R{}_{n\hat{n}}$ is approximated by the skew-symmetric matrix of the vector of orientation error Euler angles $\Psi {}_{n\hat{n}}$:

(14) $$R{}_{n\hat{n}}\approx \left(I{}_{3\times 3}+\Psi {}_{n\hat{n}}\times \right)$$

These linearized error equations depend on the current trajectory, which is the true position, velocity, orientation and the corresponding ideal inertial measurements. As we are only interested in the system’s basic response, we trade accuracy for simplicity by only looking at the most typical vehicle state and take further assumptions to eliminate the trajectory dependency and reduce complexity:

(1) The specific forces $f{}_{b}$ are selected to represent a stationary (ground) vehicle or an aircraft at straight and level flight. The only acceleration is the local gravity as measured by the accelerometer’s down-pointing *z*-axis. The local gravity measurement is approximated by the standard gravity $g_{0}$.
(2) Vertical states are omitted for this analysis. The instability of the vertical channel is well known for inertial navigation. In consequence, inertial navigation systems are almost always used with additional aiding of the vertical channel, e.g., barometric altitude measurements in aviation. This bypasses the error dynamics of the vertical channel, which motivates neglecting the corresponding states for this analysis.
(3) The meridional and normal radii $R_{M}$ and $R_{N}$ are approximated by a single Gaussian mean radius $R_{G}=\sqrt{R_{M}R_{N}}$. The maximum error arising from this approximation occurs along the equator and is only 0.3% of the true radii.
(4) The vehicle’s velocity is neglected. This eliminates any trajectory dependency and creates a more general approximation. Although the transport rate due to the vehicle’s velocity may reach the same order of magnitude as the Earth’s angular rate, the resulting Coriolis forces are usually negligible compared to e.g., the specific forces errors. Jekeli [6] states a maximum velocity of about 200 m/s up to which the vehicle’s velocity can be neglected for the error propagation without major impairs.
(5) The orientation $R{}_{nb}$ is neglected for the inputs. This is equivalent to choosing inertial measurement inputs in the local navigation frame instead of the body frame. For isotropic and uncorrelated sensor triads, the input covariance is spherical and a transformation via the orientation matrix $R{}_{nb}$ has no effect anyway on such a sphere.

Incorporating these approximations into the linearized strapdown error dynamics (11) to (13) yields the following linear state space system:

(15) $$\begin{array}{cc} \underset{\dot{z}{}_{}}{\underset{︸}{\left[\begin{array}{c} \delta \dot{\phi }\\ \delta \dot{\lambda }\\ \delta {\dot{v}}_{n}\\ \delta {\dot{v}}_{e}\\ \delta \dot{\Phi }\\ \delta \dot{\Theta }\\ \delta \dot{\Psi } \end{array}\right]}} & =\underset{A{}_{s}}{\underset{︸}{\left[\begin{array}{ccccccc} 0 & 0 & \frac{1}{R_{G}} & 0 & 0 & 0 & 0\\ 0 & 0 & 0 & \frac{1}{R_{G}\cos\phi } & 0 & 0 & 0\\ 0 & 0 & 0 & -2\omega_{ie}\sin\phi & 0 & -g & 0\\ 0 & 0 & 2\omega_{ie}\sin\phi & 0 & g & 0 & 0\\ \omega_{ie}\sin\phi & 0 & 0 & \frac{-1}{R_{G}} & 0 & -\omega_{ie}\sin\phi & 0\\ 0 & 0 & \frac{1}{R_{G}} & 0 & \omega_{ie}\sin\phi \; & 0 & \omega_{ie}\cos\phi \\ \omega_{ie}\cos\phi & 0 & 0 & \frac{\tan\phi }{R_{G}} & 0 & -\omega_{ie}\cos\phi & 0 \end{array}\right]}}\underset{z{}_{}}{\underset{︸}{\left[\begin{array}{c} \delta \phi \\ \delta \lambda \\ \delta v_{n}\\ \delta v_{e}\\ \delta \Phi \\ \delta \Theta \\ \delta \Psi \end{array}\right]}}+\\ \; & \;\underset{B{}_{s}}{\underset{︸}{\left[\begin{array}{cccccc} 0 & 0 & 0 & 0 & 0 & 0\\ 0 & 0 & 0 & 0 & 0 & 0\\ 1 & 0 & 0 & 0 & 0 & 0\\ 0 & 1 & 0 & 0 & 0 & 0\\ 0 & 0 & 0 & 1 & 0 & 0\\ 0 & 0 & 0 & 0 & 1 & 0\\ 0 & 0 & 0 & 0 & 0 & 1 \end{array}\right]}}\underset{\delta u{}_{}}{\underset{︸}{\left[\begin{array}{c} \delta f_{b,n}\\ \delta f_{b,e}\\ \delta f_{b,d}\\ \delta \omega_{ib,n}\\ \delta \omega_{ib,e}\\ \delta \omega_{ib,d} \end{array}\right]}} \end{array}$$

Despite the various assumptions, this simplified error model contains all three well-known strapdown error dynamics (Schuler, 24 h and Foucault oscillations) that are also observed in the system’s responses to sensor errors. Using the above state space system and a linear output mapping described by the matrix $C{}_{s}$:

(16) $$y{}_{}=C{}_{s}z{}_{}$$

the transfer function $G{}_{}\left(s\right)$ from sensor errors in $u{}_{}$ to selected navigation error states $y{}_{}$ of interest can be determined as [26]:

(17) $$G{}_{}\left(s\right)=C{}_{}{\left(sI{}_{}-A{}_{s}\right)}^{-1}B{}_{s}$$

Transformation of the transfer function $G{}_{}\left(s\right)$ from the frequency to the time domain yields the impulse response $g{}_{}\left(t\right)$ in the time domain:

(18) $$g{}_{}\left(t\right)=L^{-1}\left\{G{}_{}\left(s\right)\right\}$$

**Figure 1:**
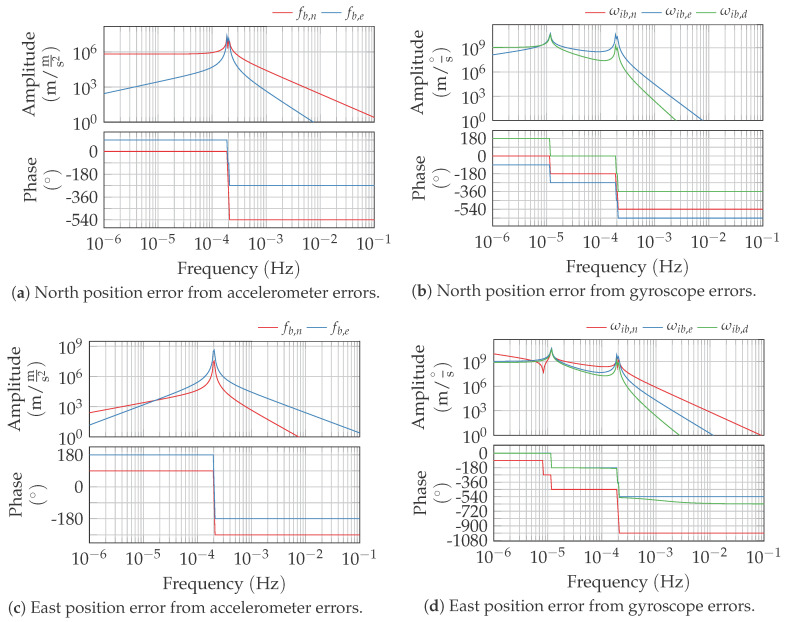
Bode plots of the strapdown error dynamics transfer functions at a geodetic latitude of 45°.

Using these equations, the transfer functions and impulse responses of the strapdown inertial error dynamics are determined. In contrast to literature [1,3,4,6], no further approximations are made. The resulting lengthy expressions are presented in Appendix A. The corresponding Bode plots for gyroscope and accelerometer inputs to the north and east position errors are depicted in Figure 1. The determined transfer functions display up to three different complex conjugate poles at:

- the Earth angular rate $\omega_{ie}$
- the rates $\omega_{s_{-}}^{2}=2\omega_{ie}^{2}\sin^{2}\phi +\omega_{s}^{2}-2\omega_{ie}\sin\phi \sqrt{\omega_{ie}^{2}\sin^{2}\phi +\omega_{s}^{2}}$
- and $\omega_{s_{+}}^{2}=2\omega_{ie}^{2}\sin^{2}\phi +\omega_{s}^{2}+2\omega_{ie}\sin\phi \sqrt{\omega_{ie}^{2}\sin^{2}\phi +\omega_{s}^{2}}$

The trigonometric addition theorem allows the interpretation of the two frequencies $\omega_{s_{-}}$ and $\omega_{s_{+}}$ as a Schuler oscillation:

(19) $$\omega_{s}=\sqrt{\frac{g_{0}}{R_{G}}}$$

that is modulated at the Foucault rate $\omega_{f}$:

(20) $$\omega_{f}=\omega_{ie}\sin\phi$$

In consistency with the literature, the positions errors follow that modulated Schuler oscillation when driven by accelerometer errors. When excited by gyroscopic errors, the additional 24 h oscillation can be observed.

### 2.2. Propagation of White Noise

Using the determined transfer functions, the strapdown system’s response to deterministic bias-like errors can be easily determined as its step response. For stochastic input, like sensor noise, the output will be stochastic and thus described by its stochastic moments, e.g., mean and variance. First, the propagation of white Gaussian sensor noise input and the navigation system’s output error variance is determined. As (15) is a (locally) linear time-invariant system, the system’s response y(t) to an input u(t) is determined from the convolution of the system’s impulse response g(t) and the input signal u(t):

(21) $$y\left(t\right)=g\left(t\right)*u\left(t\right)=\int_{0}^{t}g(\tau )u(t-\tau )d\tau$$

Using above formula, the expected value $\mu_{y}$ of the system’s response to white noise can be determined. For stationary white noise input, the expected value of the system’s output is simply the step response scaled by the input’s expected value:

(22) $$\mu_{y}\left(t\right)=E\left[y(t)\right]=E\left[\int_{0}^{t}g(\tau )u(t-\tau )d\tau \right]=\mu_{u,\nu }\int_{0}^{t}g(\tau )d\tau =\mu_{u,\nu }h\left(t\right)$$

A zero-mean white Gaussian noise input results in a zero-mean output $\mu_{y}\left(t\right)=0$. By definition, the auto covariance of white noise is zero for any two different evaluation times τ≠ρ and the variance $\sigma_{u}^{2}$ if τ=ρ. Finally, the variance of the output signal $\sigma_{y}^{2}\left(t\right)$ from white Gaussian noise input of variance $\sigma_{u}^{2}$ is determined to:

(23) $$\begin{array}{cc} \sigma_{y}^{2}\left(t\right) & =E\left[y{\left(t\right)}^{2}\right]-\mu_{y}^{2}=E\left[\int_{0}^{t}g(t-\rho )u(\rho )d\rho \int_{0}^{t}g(t-\tau )u(\tau )d\tau \right]\\ \; & =\int_{0}^{t}\int_{0}^{t}g\left(t-\rho \right)\underset{N_{u}^{2}\delta \left(\rho -\tau \right)}{\underset{︸}{E[u(\rho )u(\tau )]}}d\rho \;g\left(t-\tau \right)d\tau \\ \; & =N_{u}^{2}\int_{0}^{t}g^{2}\left(t-\tau \right)d\tau \end{array}$$

Above equation allows the determination of the navigation error’s (e.g., position) variance from white Gaussian noise on the inertial measurements inputs. In the next section, this concept is adapted to incorporate the non-white inertial sensor noise processes.

### 2.3. Propagation of Colored Sensor Noise Processes

The above-derived propagation of white Gaussian noise is enhanced to incorporate the most typical inertial sensor noise processes. These sensor noise processes are characterized by a specific shape of its power spectral density and a scaling coefficient. These coefficients are usually determined from an Allan variance analysis of the recorded sensor noise as described in [20]. Different descriptions of inertial sensor noise processes can be found in the literature, e.g., [11,27,28]. In this manuscript, we follow the definitions of the IEEE inertial sensor standards [18,19,20]. The typical noise processes and their defining properties are summarized in Table A3 in Appendix C. Although the listed processes are labeled for gyroscope measurements (angles and rates), they also apply to accelerometers: gyroscope *angular random walk* corresponds to accelerometer *velocity random walk* and analogously rate noise corresponds to acceleration noise.

For a (wide-sense) stationary stochastic process, the Wiener–Khinchin theorem states that the PSD of an output signal is the squared magnitude of the system’s transfer function $G_{P}\left(f\right)$ times the input’s PSD [29]:

(24) $$S_{y}\left(f\right)={\left|G_{P}\left(f\right)\right|}^{2}S_{x}\left(f\right)$$

**Figure 2:**
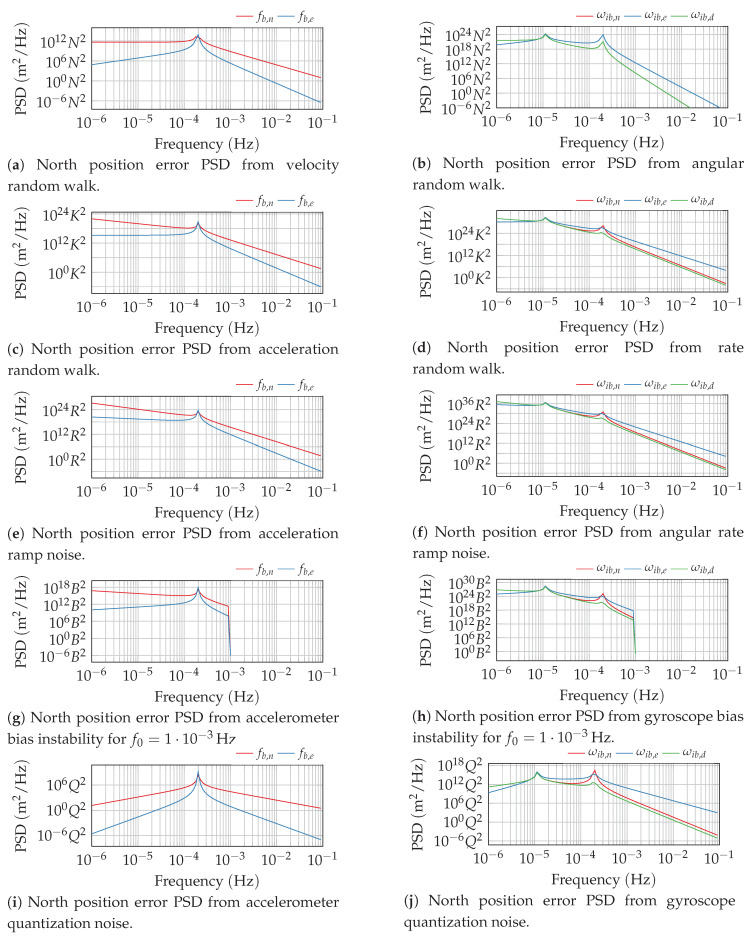
Power spectral density of the north position error from different inertial sensor noise processes.

Using the defining PSD of the different noise processes and the strapdown error dynamic’s transfer function $G_{p}\left(s\right)$, the resulting PSD of the navigator’s output error can thus be easily determined. The resulting PSD of the north position error is exemplarily depicted in Figure 2 for the different noise processes of Table A3. The strapdown error dynamics itself has a low-pass-like behavior. All non-white noise processes, except the quantization noise, excite the system dominantly in the low-frequency spectrum, which is propagated through the strapdown error dynamics. Although the quantization noise is dominated by the higher frequencies, the low-pass behavior of the strapdown dynamics still attenuates this excitation effectively.

**Figure 3:**
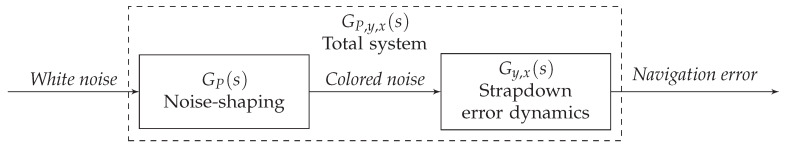
Determination of the strapdown inertial navigation system’s error response to colored noise by including a noise-shaping filter into the system’s transfer function.

The Wiener–Khinchin theorem (24) provides an option for creating colored noise from white noise input. As illustrated in Figure 3, a suitable filter with transfer function $G_{p}\left(s\right)$ can be used to create noise with the PSD of the desired noise process. Combining this transfer function with the strapdown error dynamics transfer function yields a total system that describes the navigator’s response to this particular noise process; however, it is not always possible to find such a simple filter, which requires another approach for modeling the bias instability and rate ramp noise.

#### 2.3.1. Angular Random Walk

As defined in Table A3, the angular velocity random walk is characterized by a constant PSD of amplitude $N^{2}$ and is thus simply white noise on the angular rate respectively acceleration. Integrating this noise gives a random walk process on the angle or velocity output, hence the name. The variance of an arbitrary navigation error state *y* from the input *x* is already given by (23). Introducing the angular random walk’s scaling coefficient *N* yields:

(25) $$\sigma_{N,y,x}^{2}=N^{2}\int_{0}^{t}g_{y,x}^{2}\left(t-\tau \right)d\tau$$

**Figure 4:**
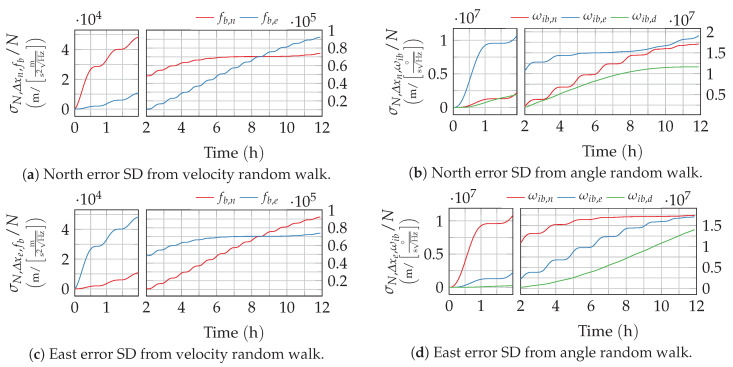
Position error growth from the angular velocity random walk noise. The time axis of the plots is split to provide a better resolution of the short-term response.

For the given sine- and cosine-based transfer functions from Appendix A, this integral can be solved analytically. The lengthy, general solution is given in Appendix B. The resulting error growth from angular velocity random walk, described here by the position errors standard deviation (SD) $\sigma_{N,\Delta x}$, is depicted in Figure 4. These curves match well with the theoretical and numerical results published by Flynn [7]. As expected, the strapdown error dynamics’ characteristic oscillations, especially the Schuler oscillation, can also be observed in the response to sensor noise.

#### 2.3.2. Rate Random Walk

Rate random walk is a random walk process on the rate measurements. Following Table A3 it is characterized by a quadratically decreasing PSD (red noise or Brownian noise). The same applies analogously to the acceleration measurement. Random walk is created from time-integration of white noise, which is given by the following transfer function:

(26) $$G_{K}\left(s\right)=K\frac{1}{s}$$

The total system’s impulse response that represents the strapdown error response to rate (or acceleration) random walk is thus simply the time integral of the strapdown navigation error’s impulse response:

(27) $$g_{K,y,x}=K\int_{0}^{t}g_{y,x}\left(\tau \right)d\tau$$

Due to their structure, the impulse responses can be easily integrated analytically. Analogous to the angular random walk, the resulting total impulse response and (23) are finally used to determine the system’s response to rate random walk noise:

(28) $$\sigma_{K,y,x}^{2}=\int_{0}^{t}g_{K,y,x}^{2}\left(t-\tau \right)d\tau$$

**Figure 5:**
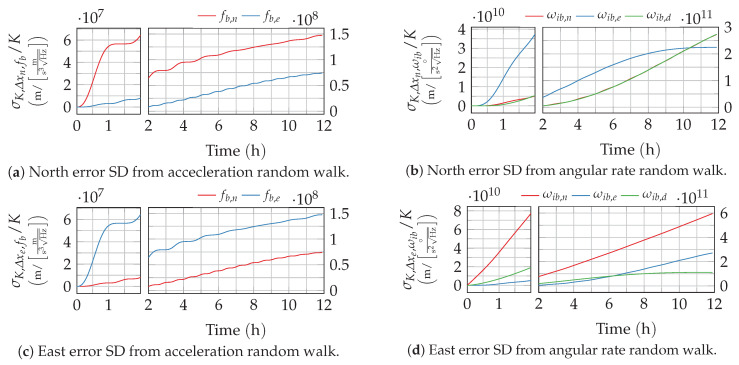
Position error growth from rate respectively acceleration random walk noise. The time axis of the plots is split to provide a better resolution of the short-term response.

Again, this integral can be solved analytically for the impulse responses using the general solution from Appendix B. The resulting position standard deviations are depicted in Figure 5.

#### 2.3.3. Quantization Noise

Quantization noise is characterized by a quadratically increasing PSD (violet noise), which corresponds to the time-derivative of white noise. The auto-covariance of such noise is given by the second time derivative of the Dirac delta function:

(29) $$E\left[u(\rho )u(\tau )\right]=\ddot{\delta }\left(\rho -\tau \right)$$

Inserting definition (29) into (23) and using the defining PSD from Table A3 the variance of the navigation error states from quantization noise is determined to:

(30) $$\sigma_{Q,y,x}^{2}\left(t\right)=Q^{2}\tau_{s}g_{y,x}^{2}\left(t\right)$$

**Figure 6:**
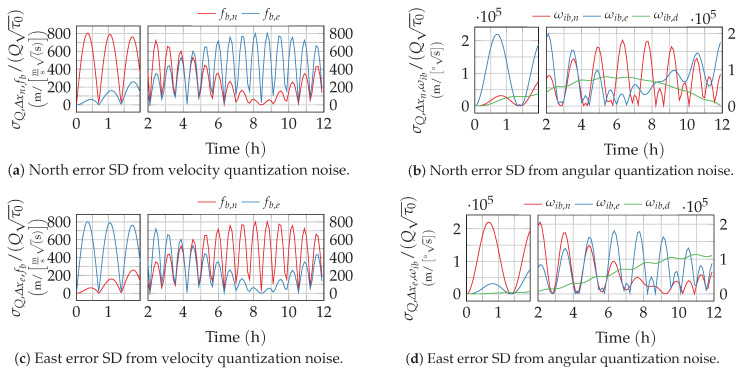
Position error growth from quantization noise. The time axis of the plots is split to provide a better resolution of the short-term response.

Note that quantization noise, in contrast to the other noise processes, scales with the sample time $\tau_{s}$. The resulting normalized position errors from gyro and accelerometer quantization noise are depicted in Figure 6. In contrast to the other noise processes, quantization noise leads to pure position oscillations and thus to a bounded position error.

An alternative analysis of inertial sensor quantization noise in strapdown navigation, especially in the context of two-speed algorithms, can be found in [10]. This paper, however, aims to stay within the noise processes framework defined by the IEEE test and specification standards.

#### 2.3.4. In-Run Bias Instability

In-run bias instability is a slow in-run variation of the sensor output’s bias. In Table A3, the bias instability is defined by a linearly decreasing PSD (flicker noise or pink noise) that is cut off hard at a frequency $f_{0,B}$. This definition poses two practical problems to the analytical approach that has been used to model the noise processes so far:

1. Generating flicker noise would require a filter with the following irrational transfer function:
  (31) $$G_{\mathrm{flicker}}\left(s\right)=\frac{1}{\sqrt{s}}$$

There is no LTI system that corresponds to such a transfer function. Although $G_{fliCke}$ can be transformed to the time-domain, the resulting impulse response

(32) $$g_{\mathrm{flicker}}\left(t\right)=\frac{1}{\sqrt{\pi t}}$$

has little use, since Equation (21) is only valid for LTI systems. Additionally, the impulse response is not even defined at time t=0.

2. Also, the theoretical hard cutoff at $f_{0,B}$ cannot be represented by a linear filter. In practice, it has to be approximated by a suitable low-pass filter.

Traditionally, flicker noise is approximated by the combination of multiple linear filters [30]. This approach, however, is only a rough approximation. The longer the signal time, the more poles are required in the filter [31]. Therefore, another approach is used in this manuscript. As stated above, the impulse response in continuous time cannot be used for the analysis. However, a method proposed by Kasdin [31] is used to create a time-discrete impulse response that accurately represents power law noise of the full time range of interest. This impulse response used to create power law noise of PSD $1/f^{\alpha }$ is defined recursively:

(33) $$\begin{array}{cc} g_{\alpha }\left[0\right] & =1 \end{array}$$

(34) $$\begin{array}{cc} g_{\alpha }\left[k\right] & =\left(k-1-\frac{\alpha }{2}\right)\frac{g[k-1]}{k} \end{array}$$

Note that this discrete-time impulse response is not a time-discretization of the theoretical continuous time impulse response but specifically designed to create a power-law noise sequence that has the desired PSD and auto-correlation.

**Figure 7:**
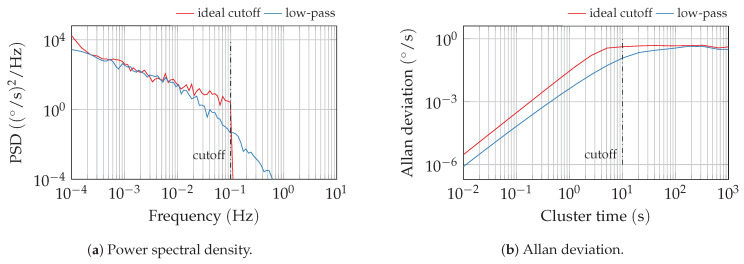
Comparison of the power spectral density (PSD) and Allan variance of simulated bias instability signals for a sharp cutoff and first-order low-pass approximation.

For this analysis, the cutoff in the bias instability is approximated by a first-order low-pass filter. A comparison of bias instability signals with a sharp cutoff and this approximation is depicted in Figure 7. This approximation shifts the Allan variance slope slightly to higher cluster times but the level of the plateau is virtually unchanged. A reduction of the cutoff time $T_{0,B}$ by a factor of about 1/3 compared to the identified cutoff yields a good approximation in simulation (see Section 3.2). Nevertheless, in practice, the high-frequency parts of sensor noise are highly dominated by other processes such as angular random walk. Consequently, the inaccurate PSD slope beyond the cutoff frequency is covered by the other processes.

With the impulse response of flicker noise, which is Equation (34) for α=1, and the following impulse response of a first order low-pass filter with time constant $T_{0,B}$

(35) $$\begin{array}{cc} g_{lp}\left[k\right] & =g_{lp}\left[k-1\right]\left(1-\frac{\tau_{s}}{T_{0,B}+\tau_{s}}\right) \end{array}$$

(36) $$\begin{array}{cc} g_{lp}\left[0\right] & =\frac{\tau_{s}}{T_{0,B}+\tau_{s}} \end{array}$$

the final impulse response of a fictive filter that generates bias instability noise can be determined to:

(37) $$g_{B}\left[k\right]=\sum_{l=0}^{k-1}g_{\alpha =1}\left[k-l\right]g_{l}p\left[l\right]$$

The total system impulse response for bias instability excitation can then be determined using the discrete-time version of (21):

(38) $$g_{B,y,x}\left[k\right]=B\sum_{l=0}^{k-1}g_{y,x}\left[k-l\right]g_{B}\left[l\right]$$

The output’s variance at time *k* is finally determined from [31]:

(39) $$\sigma_{B,y,x}^{2}\left[k\right]=\sum_{l=0}^{k-1}g_{B,y,x}^{2}\left[l\right]$$

**Figure 8:**
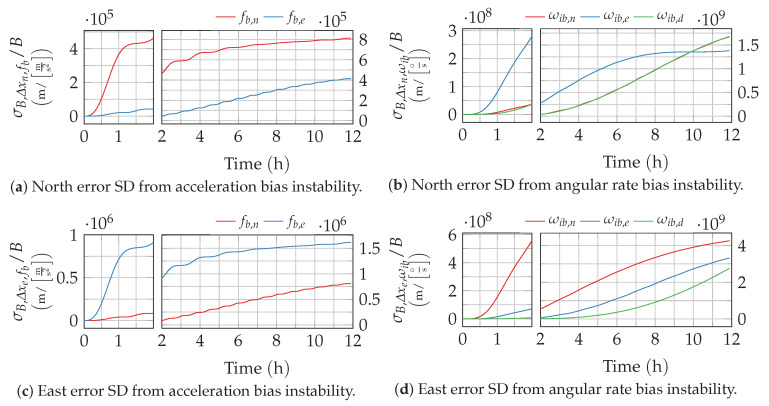
Position error growth from bias instability with $T_{0}=5000\;s$. The time axis of the plots is split to provide a better resolution of the short-term response.

**Figure 9:**
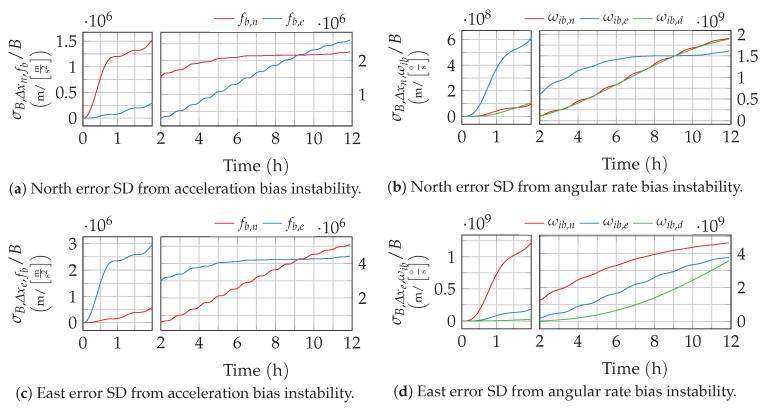
Position error growth from bias instability with $T_{0}=100\;s$. The time axis of the plots is split to provide a better resolution of the short-term response.

Clearly, the resulting response depends on the selected cutoff frequency $f_{0,B}$ (respectively time constant $T_{0,B}=1/f_{0,B}$). The resulting growth of the position uncertainty is depicted by way of example in Figure 8 and Figure 9 for different values of $T_{B}$. Higher time constants, like 5000s, that are typical for optical gyroscopes lead to a slower growth in the short term and a reduction of the Schuler oscillation amplitudes. For the low time of about 100 s, which is more representative for accelerometers, the Schuler oscillations are still clearly visible on top of the long-term error growth. Both observations match with the low-pass-like shape of the bias instability’s PSD.

For assessing and predicting the influence of bias instability on the navigation error, not only the scaling *B* (which may be given in data sheets), but also the time constant $T_{0,B}$, or at least its approximate magnitude, is required. Both can be determined from e.g., Allan variance charts of the sensor’s noise.

#### 2.3.5. Rate Ramp Noise

As summarized in Table A3, rate ramp noise, respectively acceleration ramp noise, is characterized by a PSD that declines cubically with the frequency. From this definition, the shaping filter’s transfer function can be easily determined to:

(40) $$G_{R}\left(s\right)=\frac{R}{{\left(2\pi f\right)}^{3/2}}$$

**Figure 10:**
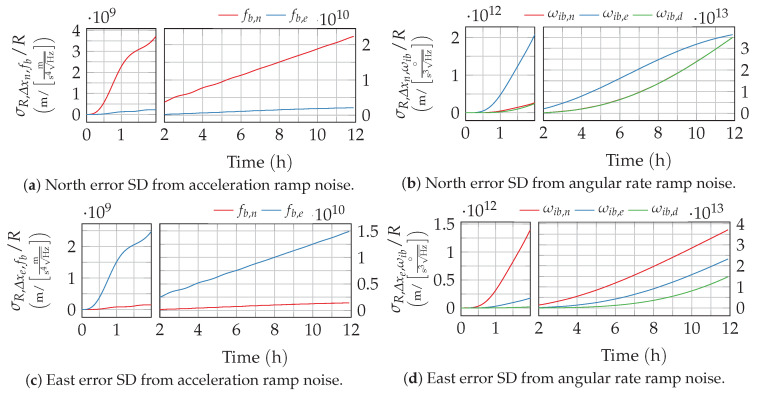
Position error growth from rate ramp noise. The time axis of the plots is split to provide a better resolution of the short-term response.

Analogous to the bias instability, this irrational transfer function cannot be handled with the continuous-time approach. However, the already introduced Equation (34) directly yields the discrete-time impulse response $g_{R}\left[k\right]$ that shapes rate ramp noise from white noise for α=3. From that, the total system response is then determined to:

(41) $$g_{R,y,x}\left[k\right]=R\sum_{l=0}^{k-1}g_{y,x}\left[k-l\right]g_{R}\left[l\right]$$

and finally used in

(42) $$\sigma_{R,y,x}^{2}\left[k\right]=\sum_{l=0}^{k-1}g_{R,y,x}^{2}\left[l\right]$$

to determine the navigation error variance from rate ramp or acceleration ramp noise. The resulting position error standard deviation over time is depicted in Figure 10. As rate ramp noise is dominated by low-frequency parts, the position error growths show even less dynamics than the bias instability.

## 3. Results

### 3.1. Predicting Strapdown Inertial Navigation Performance

The different responses of the strapdown error dynamics to excitation by different noise processes have been derived in the previous section. Once the different noise process parameters of the inertial measurement unit (IMU) are identified (or taken from a data-sheet), the derived solutions can be easily used to determine the position variance from each single sensor axis and noise process. This can be performed by implementing and evaluating the derived Equations (25), (28), (30), (39) and (42) with the appropriate transfer functions from Appendix A in a suitable programming environment. Analytical solutions for the integrals can be found in Appendix B. Alternatively, the position variance at a given time can be simply read off of the charts provided in Figure 4, Figure 5, Figure 6, Figure 7, Figure 8, Figure 9 and Figure 10 and scaled with the respective noise parameters.

For the linearized error dynamics, the total position variances can be easily determined by adding up the variances of the different processes and axes $j\in (\omega_{ib,x},\omega_{ib,y},\omega_{ib,z},f_{b,x},f_{b,y},f_{b,z})$. For the north position error this yields:

(43) $$\begin{array}{cc} \sigma_{\Delta x_{n}}^{2}\left(t\right) & =\sum_{j}\sigma_{N,\Delta x_{n},j}^{2}\left(N_{j},t\right)+\sum_{j}\sigma_{K,\Delta x_{n},j}^{2}\left(K_{j},t\right)+\sum_{j}\sigma_{Q,\Delta x_{n},j}^{2}\left(Q_{j},t\right)+\\ & \;\sum_{j}\sigma_{B,\Delta x_{n},j}^{2}\left(B_{j},t\right)+\sum_{j}\sigma_{R,\Delta x_{n},j}^{2}\left(R_{j},t\right) \end{array}$$

The east position variance $\sigma_{\Delta x_{e}}^{2}\left(t\right)$ is determined analogously. As the resulting position errors are zero-mean they can be easily combined into a single measure, e.g., distance root mean square (DRMS):

(44) $$\mathrm{DRMS}\left(t\right)=\sqrt{\sigma_{\Delta x_{n}}^{2}\left(t\right)+\sigma_{\Delta x_{e}}^{2}\left(t\right)}$$

In addition to the navigation errors from sensor noise presented here, the position errors from e.g., sensors biases and navigation state initialization errors should be considered in the sensor selection process. A discussion of these errors can be found in classic literature, e.g., [2,3,4]. The presented method for predicting the positional uncertainty growth is best understood from the following example.

### 3.2. Example: Navigation Error Prediction for a Fiber Optic Gyroscope IMU

In the following example, we demonstrate the approximation of the navigation errors from sensor noise of an exemplary FOG IMU. The different noise process parameters were identified from the Allan Variance analysis of a 48 h recording of the stationary IMU. The noise coefficients were determined from a least-squares fit of the IEEE noise process models to the Allan variance curve that was determined from the recorded sensor noise, as suggested in [32]. The identified parameters are summarized in Table 1.

**Figure 11:**
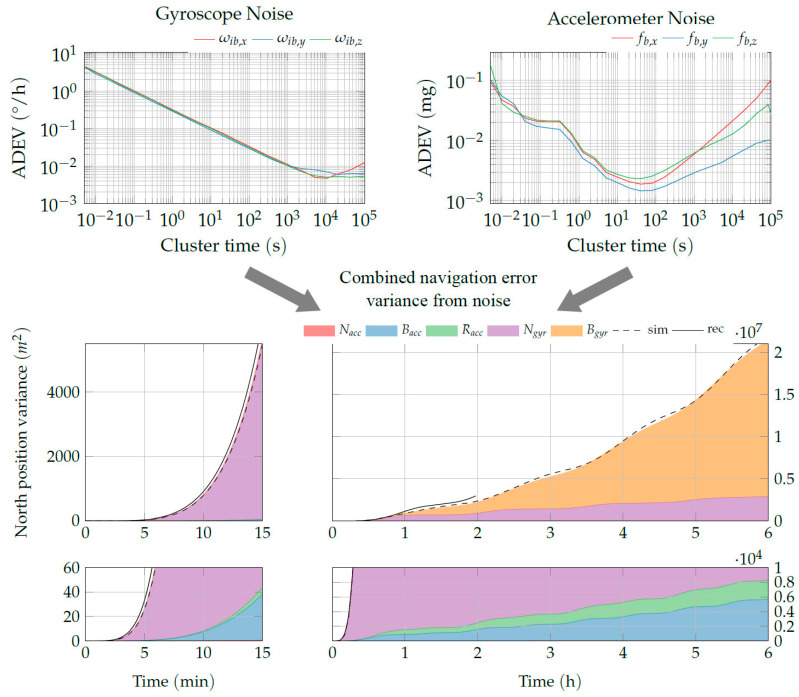
Composition of the north position variance for example noise processes identified from a Fiber Optic Gyroscope compared to the resulting variance from 10,000 Monte Carlo runs with numerically simulated synthetic IMU noise (labeled sim, dashed line) and 24 runs using the real recorded IMU noise (labeled rec, solid line).

As described in Section 3.1, the formulas for the position error variance from Section 2.3 were implemented and evaluated in Matlab R2019b. For example, the results for the north position error variance are depicted in Figure 11. The respective contributions from the different noise processes are represented by the colored faces that add up to the total north position error variance.

To account for the low-pass approximation of the bias-instability (see Figure 7), the cutoff time $T_{0,B}$ used in the analytical solution is reduced to 1/3 of the identified time. This approximation yields good results compared to the numerical simulation with a hard cutoff of the bias instability. The result of 10,000 Monte Carlo runs with numerically generated IMU noise in the full non-linear strapdown navigation is added for comparison. Here, one advantage of the analytical approach becomes obvious: The numerical evaluation of the derived expressions requires only 0.6 s, whereas the Monte Carlo simulation takes 11 h on an average desktop computer. Additionally, the resulting variance from multiple strapdown navigation simulation runs using the real recorded sensor outputs is depicted. The 48 h recorded IMU data are split into 24 chunks of 2 h each, to allow multiple simulation runs. Both results fit the analytically predicted variance well. The small deviation of the recorded data can be explained by the low number of iterations with the recorded data as well as additional factors like alignment and initialization errors.

For the utilized IMU, the navigation error is clearly dominated by the gyroscope errors. In particular, the gyro angular random walk dominates the short-term errors. Starting at about 90 min, the gyro bias instability surpasses all other error sources. For the accelerometers, only the low-frequency errors (bias instability and acceleration ramp) are relevant. Still, the errors from gyroscope noise are several magnitudes higher for this IMU configuration.

### 3.3. Conditions for Neglecting Colored Sensor-Noise

The previous example clearly shows how the different noise processes contribute differently to the overall position error at different times. For short times, the position error is clearly dominated by the gyro angular random walk, whereas this gradually changes in favor of the bias instability. For the given example, the typical approach to model the sensor’s noise as simple white noise on the rate output (angular random walk only) seems justified for at least the first 30 min of propagation.

To obtain a more general statement, we look at the ratios of the position errors caused by the different noise processes. The total position error (DRMS) caused by a specific noise process shall be only a fraction *k* of the position error caused by the angular or velocity random walk. To be consistent with practice, we can assume equal noise coefficients for all axis of the sensor triads. For the gyro bias instability, this condition yields, for example:

(45) $$\begin{array}{cc} {\mathrm{DRMS}}_{B}\left(B_{\omega_{ib}},t\right) & \leq k\;\cdot \;{\mathrm{DRMS}}_{N}\left(N_{\omega_{ib}},t\right) \end{array}$$

(46) $$\begin{array}{cc} B & \leq kN\;\cdot \;\frac{\sqrt{\left(\sigma_{\Delta n,N}^{2}\left(t\right)+\sigma_{\Delta e,N}^{2}\left(t\right)\right)}}{\sqrt{\left(\sigma_{\Delta n,B}^{2}\left(t\right)+\sigma_{\Delta e,B}^{2}\left(t\right)\right)}} \end{array}$$

**Figure 12:**
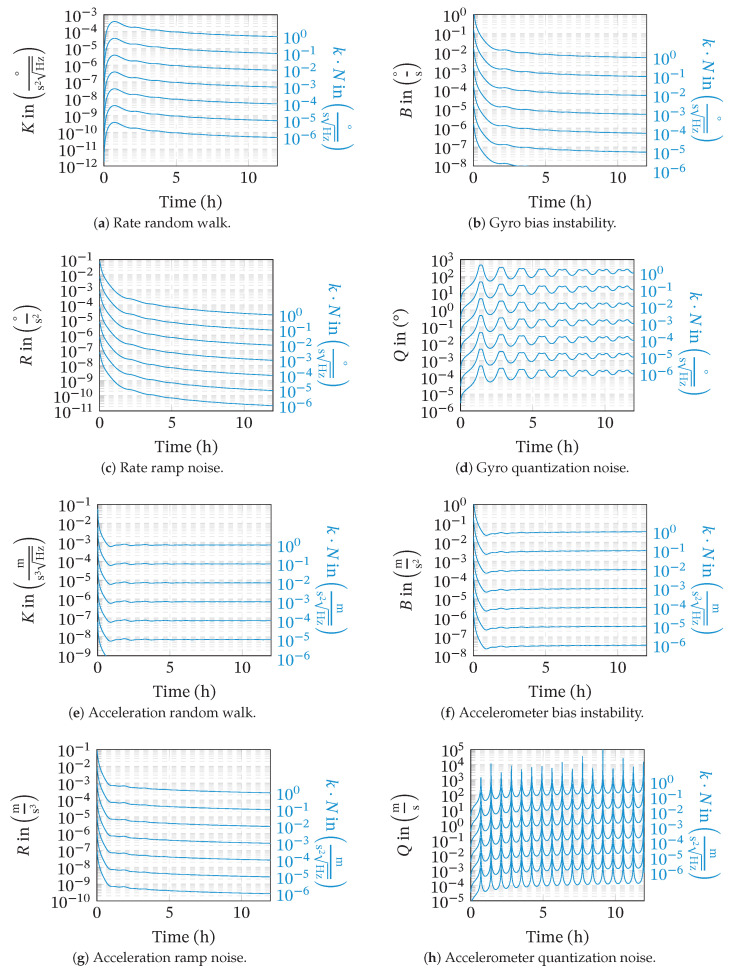
Maximum noise coefficients for scaled angular/velocity random walk coefficient *kN* over time. Graphs were determined for a latitude of 45° and an altitude of 0 m. For a given time *t*, the lines indicate the noise coefficients where e.g., the bias instability’s contribution to the position uncertainty is a fraction *k* of the angular random walk’s contribution.

The resulting maximum noise coefficients for rate random walk, bias instability, rate ramp noise, quantization noise and their accelerometer counterparts are depicted in Figure 12. The charts can be used as follows:

(1) Choose the maximum ratio *k* of the position error (DRMS) caused by the colored noise process and the DRMS caused by angular or velocity random walk, e.g., k=0.01.
(2) For a given angular random walk coefficient *N*, find the blue plot line closest to k·N.
(3) Read off the maximum acceptable noise coefficient, e.g., *B*, at the desired time. The selected coefficients now fulfill Equation (45) at time *t*.

In contrast to the other noise processes, which are usually hard to identify or are not observed at all, the bias instability and angular random walk can be observed for virtually every inertial sensor. Using above described method, the maximum mission time that allows for the neglect of the bias instability compared to the angular random walk is summarized for several sensors in Table 2. The sensors were chosen based on their publicly available Allan variance plots to represent a wide range of gyroscope grades. The given DRMS values give the total position error from bias instability and angular random walk, only. Further sensor errors are not considered in this analysis.

In general, higher sensor grades provide better long-term stability, but this does not allow a statement on the maximum acceptable time for neglecting the bias instability since this depends on the ratio of the bias instability and the angular random walk. For the FOG gyro DSP3100, for example, we determined a threshold of 55 s, whereas the MEMS based STIM300 allows the bias instability to be neglected up to a time of 96 s. Still, the FOG gyro’s position drift is one magnitude better than the MEMS-based example.

Even for low-cost sensors, the bias instability contributes significantly to the total position error only after several seconds. This gives a hint regarding the necessity of considering the bias instability when modeling the sensor noise in certain applications. The free inertial propagation time between two position fixes in an integrated navigation system is usually below 1 s. Even when considering short outages of the satellite navigation systems (GNSS), the bias instability will not contribute significantly to the position growth within this time scale. Navigation-grade sensors, however, are used to provide unaided position reference for hours or longer. For these time scales, the bias instability clearly yields a significant contribution to the position error and should be considered in the analysis.

Similarly, the other $1/f^{\alpha }$ noise processes become significant for long-term navigation only. As illustrated in Figure 12, the contribution of the quantization noise is worst for short times. Still, from the charts, it can be determined whether a certain level of quantization noise can be neglected, independent of the mission duration.

## 4. Limitations

The derived analytical solutions and charts provide an easy-to-use method to estimate the strapdown navigation errors caused by different inertial sensor noise processes. This simplicity comes with the caveat of extensive assumptions on the vehicle’s dynamics and the sensor’s behavior:

- The analytical solutions are only valid for a stationary vehicle. The accuracy of the error dynamic’s approximation decreases with the actual velocity.
- The vehicle is assumed to be straight and leveled.
- The navigation system’s vertical channel is fixed by an external aiding.
- The position errors must be kept below about 100 km to stay within the valid region of the linearized error dynamics.
- The sensor’s noise characteristics are assumed to be constant. They neither depend on the time nor the trajectory.
- The presented graphs were created for a latitude of 45 ${}^{{}^{\circ}}$. Of course, the analytical solution allows for a simple evaluation at any other latitude more representative for a certain application.

Given the above limitations, the described method can only provide qualitative statements and no definite prediction of the navigation errors. Of course, all of these assumptions could be easily abandoned in a Monte Carlo simulation to generate a quantitative prediction. This, however, requires detailed error models and a known mission trajectory, which is usually not available at an early stage of development. In this case, the developed method allows for an early assessment of the suitability of different sensors. Within this manuscript, we considered only noise-like sensor errors, but uncompensated bias-like errors typically result in higher navigation errors. The presented methods should therefore be combined with the results for bias-like errors that can be found in the literature [2,3,4] to get a complete picture.

## 5. Conclusions

In this manuscript, we presented a method to analytically predict the position errors from colored sensor noise in strapdown inertial navigation systems. Together with literature methods for biases and initialization errors, the presented scheme allows for a simple evaluation of an inertial sensor’s navigation performance at an early design phase. Compared to Monte Carlo simulations, the method requires significantly reduced implementation effort and computing time. Additionally, the method supports the assessment of the contributions of individual noise processes and thus allows the identification of critical performance parameters in the sensor selection process. This was demonstrated for real sensor data in Section 3.2. In addition to the position errors, the presented approach can be easily adapted to the other navigation states, e.g., the orientation angles.

Due to the low-pass behavior of the strapdown inertial navigation algorithms, the impact of colored sensor noise processes, except for the quantization noise, grows with the mission time. For short times, the position uncertainty is always dominated by the white noise parts (angular or velocity random walk). The maximum time for which the white noise dominates and the other noise processes can be neglected can be easily read off of the charts provided in Figure 12. The presented examples indicate that even for low-cost sensors, it takes several seconds of propagation until the gyro bias instability contributes significantly to the position uncertainty. For integrated navigation, where the time between two consecutive updates is below 1 s, the white noise is clearly dominant. For long-term inertial navigation, however, our results clearly point out the necessity of modeling and considering all noise processes properly. In general, the focus on white sensor noise seems to be justified, but should be checked individually for each sensor configuration and mission.

## Figures and Tables

**Table 1:** Noise parameters identified from 48 h recorded data of an IFOS-500 inertial measurement unit (IMU).

	Gyro x	Gyro y	Gyro z	Acc x	Acc y	Acc z
*N*	0.0049 $\frac{\circ }{\sqrt{h}}$	0.0052 $\frac{\circ }{\sqrt{h}}$	0.0054 $\frac{\circ }{\sqrt{h}}$	0.0056 $\frac{mg}{\sqrt{Hz}}$	0.0070 $\frac{mg}{\sqrt{Hz}}$	0.0057 $\frac{mg}{\sqrt{Hz}}$
*B*	0.013 $\frac{\circ }{\sqrt{h}}$	0.0075 $\frac{\circ }{\sqrt{h}}$	0.0079 $\frac{\circ }{\sqrt{h}}$	0.0025 mg	0.0042 mg	0.0029 mg
$T_{0,B}$	3000 s	3000 s	3000 s	50 s	50 s	50 s
*R*	-	-	-	$0.55\cdot 10^{-6}$ mg Hz	$0.96\cdot 10^{-6}$ mg Hz	$0.37\cdot 10^{-6}$ mg Hz

**Table 2:** Maximum mission time that allows for the neglect of the gyro bias instability for different sensor grades. Below the threshold time, the bias instability’s contribution to the total position error is less than 1% of the angular random walk’s contribution.

			Noise Parameters				1% Threshold	
Grade	Example	Tech.	*N*	*B*	$T_{B}$	DRMS (1 h)	Time	DRMS
			$\left(\frac{{}^{\circ }}{\sqrt{h}}\right)$	$\left(\frac{{}^{\circ }}{h}\right)$	(s)	(km)	(s)	(m)
Industrial	DMU10 [33]	CVG	0.4	15	500	*2800* *	**11**	0.14
	STIM300 [34]	CVG	0.15	0.5	1000	80	**96**	12
Tactical	DSP3100 [35]	FOG	0.048	0.072	2000	7	**55**	0.1
Navigation	GG1320 [36]	RLG	0.0015	0.0024	2000	0.4	**245**	1.3

* Value clearly exceeds validity range of the linearized model.

## Abbreviations

The following abbreviations are used in this manuscript:

CVG	Coriolis Vibratory Gyroscope
DRMS	Distance Root Mean Square
ECEF	Earth-Centered Earth-Fixed Coordinate System
FOG	Fiber Optic Gyroscope
GNSS	Global Navigation Satellite System
IMU	Inertial Measurement Unit
IEEE	Institute of Electrical and Electronics Engineers
MEMS	Micro-Electro-Mechanical Systems
NED	North–East–Down Coordinate System
ODE	Ordinary Differential Equation
PSD	Power Spectral Density
RLG	Ring Laser Gyroscope
SD	Standard Deviation
**Symbols**	
α	Exponent of $1/f^{\alpha }$ PSD noise
$A{}_{s}$	System matrix of the linearized strapdown error dynamics
$b{}_{a}$	Vector of accelerometer biases
$b{}_{g}$	Vector of gyroscope biases
*B*	Bias instability coefficient
$B{}_{s}$	Input matrix of the linearized strapdown error dynamics
$C{}_{s}$	Output matrix of the linearized strapdown error dynamics
*f*	Frequency
$f{}_{b}$	IMU’s specific forces (accelerations) vector
$\gamma {}_{n}$	Local gravity vector in NED frame
$g_{0}$	Standard gravity, 9.80665 m/s${}^{2}$
g(t)	Transfer function/impulse response in the time domain
G(s)	Transfer function in the Laplace domain
*h*	Geodetic altitude
$I{}_{}$	Identity matrix
*K*	Rate/acceleration noise coefficient
λ	Geodetic longitude
$\lambda {}_{}$	Vector of geodetic position components
μ	Expected value
$M{}_{a}$	Accelerometer misalignment and scale factor matrix
$M{}_{g}$	Gyroscope misalignment and scale factor matrix
$\nu {}_{a}$	Vector of accelerometer noise
$\nu {}_{g}$	Vector of gyroscope noise
*N*	Angular/velocity random walk noise coefficient
$\omega_{s}$	Angular frequency of the Schuler oscillation
$\omega {}_{ib}$	IMU’s angular rate vector
$\omega {}_{ie}$	Vector of the Earth’s angular rate, expressed in the ECEF frame
$\omega {}_{en}$	Vector of the angular rate between the local NED and the ECEF frame expressed in the NED frame

$\Omega_{xy}$	Skew symmetric matrix of angular rate vector $\omega {}_{xy}$
*Q*	Quantization noise coefficient
ϕ	Geodetic latitude
$\psi {}_{n\hat{n}}$	Vector of orientation Euler angles representing the orientation error
*R*	Rate ramp/acceleration ramp noise coefficient
$R_{G}$	Gaussian mean of the local Earth radii
$R_{M}$	Local meridional radius of the Earth curvature
$R_{N}$	Local normal radius of the Earth curvature
$R{}_{nb}$	Rotation matrix from the body fixed frame to the local NED frame.
$R{}_{ne}$	Rotation matrix from the ECEF frame to the local NED frame.
$R{}_{n\hat{n}}$	Rotation matrix representing the orientation error of the body fixed frame with respect to the NED frame
σ	Standard deviation, root variance
S(f)	Power spectral density
*t*	Time
$T_{0,B}$	Time constant of low-pass filter used to model the in-run bias instability cut-off
$v{}_{n}$	Velocity vector in NED-frame
$v_{n}$	North velocity component
$v_{e}$	East velocity component
$v_{d}$	Down velocity component
$y{}_{}$	Output vector of the linearized strapdown error dynamics

## Appendix A. Transfer Functions

The transfer functions and corresponding impulse responses of the strapdown inertial navigation error states to inertial measurement errors are summarized in the following tables.

**Table A1:** Latitude error impulse responses.

Input	Transfer Function/Impulse Response
$\delta f_{b,n}$	$$\begin{array}{cc} G_{1,1}\left(s\right) & =\frac{\frac{1}{R}s^{2}}{\left(s^{2}+\omega_{s_{-}}^{2}\right)\left(s^{2}+\omega_{s_{+}}^{2}\right)}+\frac{\frac{\omega_{s}^{2}}{R}}{\left(s^{2}+\omega_{s_{-}}^{2}\right)\left(s^{2}+\omega_{s_{+}}^{2}\right)}\\ g_{1,1}\left(t\right) & =\left(\frac{\omega_{s_{-}}^{2}-\omega_{s}^{2}}{\omega_{s_{-}}\left(\omega_{s_{-}}^{2}-\omega_{s_{+}}^{2}\right)}\sin\left(\omega_{s_{-}}t\right)+\frac{\omega_{s_{+}}^{2}-\omega_{s}^{2}}{\omega_{s_{+}}\left(\omega_{s_{+}}^{2}-\omega_{s_{-}}^{2}\right)}\sin\left(\omega_{s_{+}}t\right)\right)\frac{1}{R} \end{array}$$
$\delta f_{b,e}$	$$\begin{array}{cc} G_{1,2}\left(s\right) & =\frac{\frac{2}{R}\omega_{ie}\sin\tilde{\phi }s}{\left(s^{2}+\omega_{s_{-}}^{2}\right)\left(s^{2}+\omega_{s_{+}}^{2}\right)}\\ g_{1,2}\left(t\right) & =\left(\frac{1}{\left(\omega_{s_{-}}^{2}-\omega_{s_{+}}^{2}\right)}\sin\left(\omega_{s_{-}}t\right)+\frac{1}{\left(\omega_{s_{+}}^{2}-\omega_{s_{-}}^{2}\right)}\sin\left(\omega_{s_{+}}t\right)\right)\frac{2}{R}\omega_{ie}\sin\tilde{\phi } \end{array}$$
$\delta f_{b,d}$	$$\begin{array}{cc} G_{1,3}\left(s\right) & =0\\ g_{1,3}\left(t\right) & =0 \end{array}$$
$\delta \omega_{ib,n}$	$$\begin{array}{cc} G_{1,4}\left(s\right) & =-\frac{3\omega_{s}^{2}\omega_{ie}\sin\tilde{\phi }s}{\left(s^{2}+\omega_{ie}^{2}\right)\left(s^{2}+\omega_{s_{-}}^{2}\right)\left(s^{2}+\omega_{s_{+}}^{2}\right)}-\frac{\omega_{s}^{2}\omega_{ie}\sin\tilde{\phi }\left(\omega_{s}^{2}-2\omega_{ie}\cos^{2}\tilde{\phi }\right)}{\left(s^{2}+\omega_{ie}^{2}\right)\left(s^{2}+\omega_{s_{-}}^{2}\right)\left(s^{2}+\omega_{s_{+}}^{2}\right)}\\ g_{1,4}\left(t\right) & =\left(\frac{3\omega_{s_{-}}^{2}-2\omega_{ie}^{2}\cos\tilde{\phi }-\omega_{s}^{2}}{\omega_{s_{-}}\left(\omega_{s_{-}}^{2}-\omega_{s_{+}}^{2}\right)\left(\omega_{s_{-}}^{2}-\omega_{ie}^{2}\right)}\sin\left(\omega_{s_{-}}t\right)+\frac{3\omega_{s_{+}}^{2}-2\omega_{ie}^{2}\cos\tilde{\phi }-\omega_{s}^{2}}{\omega_{s_{+}}\left(\omega_{s_{+}}^{2}-\omega_{s_{-}}^{2}\right)\left(\omega_{s_{+}}^{2}-\omega_{ie}^{2}\right)}\sin\left(\omega_{s_{+}}t\right)\right.\\ \; & \;\left.+\frac{3\omega_{ie}^{2}-2\omega_{ie}^{2}\cos\tilde{\phi }-\omega_{s}^{2}}{\omega_{ie}\left(\omega_{ie}^{2}-\omega_{s_{-}}^{2}\right)\left(\omega_{ie}^{2}-\omega_{2}^{2}\right)}\sin\left(\omega_{ie}t\right)\right)\omega_{s}^{2}\omega_{ie}\sin\tilde{\phi } \end{array}$$
$\delta \omega_{ib,e}$	$$\begin{array}{cc} G_{1,5}\left(s\right) & =-\frac{\omega_{s}s^{3}}{\left(s^{2}+\omega_{ie}^{2}\right)\left(s^{2}+\omega_{s_{-}}^{2}\right)\left(s^{2}+\omega_{s_{+}}^{2}\right)}-\frac{\omega_{s}^{2}\left(\omega_{s}^{2}-2\omega_{ie}^{2}\sin^{2}\tilde{\phi }\right)s}{\left(s^{2}+\omega_{ie}^{2}\right)\left(s^{2}+\omega_{s_{-}}^{2}\right)\left(s^{2}+\omega_{s_{+}}^{2}\right)}\\ g_{1,5}\left(t\right) & =\left(\frac{\omega_{s_{-}}^{2}+2\omega_{ie}^{2}\cos\tilde{\phi }-\omega_{s}^{2}}{\left(\omega_{s_{-}}^{2}-\omega_{s_{+}}^{2}\right)\left(\omega_{s_{-}}^{2}-\omega_{ie}^{2}\right)}\cos\left(\omega_{s_{-}}t\right)+\frac{\omega_{s_{+}}^{2}+2\omega_{ie}^{2}\cos\tilde{\phi }-\omega_{s}^{2}}{\left(\omega_{s_{+}}^{2}-\omega_{s_{-}}^{2}\right)\left(\omega_{s_{+}}^{2}-\omega_{ie}^{2}\right)}\cos\left(\omega_{s_{+}}t\right)\right.\\ \; & \;\left.+\frac{\omega_{ie}^{2}+2\omega_{ie}^{2}\cos\tilde{\phi }-\omega_{s}^{2}}{\left(\omega_{ie}^{2}-\omega_{s_{-}}^{2}\right)\left(\omega_{ie}^{2}-\omega_{2}^{2}\right)}\cos\left(\omega_{ie}t\right)\right)\omega_{s}^{2} \end{array}$$
$\delta \omega_{ib,d}$	$$\begin{array}{cc} G_{1,6}\left(s\right) & =-\frac{\omega_{s}^{2}\omega_{ie}\cos\tilde{\phi }s^{2}}{\left(s^{2}+\omega_{ie}^{2}\right)\left(s^{2}+\omega_{s_{-}}^{2}\right)\left(s^{2}+\omega_{s_{+}}^{2}\right)}-\frac{\omega_{s}^{2}\omega_{ie}\cos\tilde{\phi }\left(\omega_{s}^{2}-2\omega_{ie}^{2}\sin^{2}\tilde{\phi }\right)}{\left(s^{2}+\omega_{ie}^{2}\right)\left(s^{2}+\omega_{s_{-}}^{2}\right)\left(s^{2}+\omega_{s_{+}}^{2}\right)}\\ g_{1,6}\left(t\right) & =\left(\frac{3\omega_{s_{-}}^{2}-2\omega_{ie}^{2}\cos\tilde{\phi }-\omega_{s}^{2}}{\omega_{s_{-}}\left(\omega_{s_{-}}^{2}-\omega_{s_{+}}^{2}\right)\left(\omega_{s_{-}}^{2}-\omega_{ie}^{2}\right)}\sin\left(\omega_{s_{-}}t\right)+\frac{3\omega_{s_{+}}^{2}-2\omega_{ie}^{2}\cos\tilde{\phi }-\omega_{s}^{2}}{\omega_{s_{+}}\left(\omega_{s_{+}}^{2}-\omega_{s_{-}}^{2}\right)\left(\omega_{s_{+}}^{2}-\omega_{ie}^{2}\right)}\sin\left(\omega_{s_{+}}t\right)\right.\\ \; & \;\left.+\frac{3\omega_{ie}^{2}-2\omega_{ie}^{2}\cos\tilde{\phi }-\omega_{s}^{2}}{\omega_{ie}\left(\omega_{ie}^{2}-\omega_{s_{-}}^{2}\right)\left(\omega_{ie}^{2}-\omega_{2}^{2}\right)}\sin\left(\omega_{ie}t\right)\right)\omega_{s}^{2}\omega_{ie}\sin\tilde{\phi } \end{array}$$

**Table A2:** Longitude error impulse responses.

Input	Transfer Function/Impulse Response
$\delta f_{b,n}$	$$\begin{array}{cc} G_{2,1}\left(s\right) & =\frac{\frac{2}{R}\omega_{ie}\tan\tilde{\phi }s}{\left(s^{2}+\omega_{s_{-}}^{2}\right)\left(s^{2}+\omega_{s_{+}}^{2}\right)}\\ g_{2,1}\left(t\right) & =\left(\frac{1}{\left(\omega_{s_{-}}^{2}-\omega_{s_{+}}^{2}\right)}\cos\left(\omega_{s_{-}}t\right)+\frac{1}{\left(\omega_{s_{+}}^{2}-\omega_{s_{-}}^{2}\right)}\cos\left(\omega_{s_{+}}t\right)\right)\frac{2}{R}\omega_{ie}\tan\tilde{\phi } \end{array}$$
$\delta f_{b,e}$	$$\begin{array}{cc} G_{2,2}\left(s\right) & =\frac{\frac{1}{R\cos\tilde{\phi }}s^{2}}{\left(s^{2}+\omega_{s_{-}}^{2}\right)\left(s^{2}+\omega_{s_{+}}^{2}\right)}+\frac{\frac{\omega_{s}^{2}}{R\cos\tilde{\phi }}}{\left(s^{2}+\omega_{s_{-}}^{2}\right)\left(s^{2}+\omega_{s_{+}}^{2}\right)}\\ g_{2,2}\left(t\right) & =\left(\frac{\omega_{s_{-}}^{2}-\omega_{s}^{2}}{\omega_{s_{-}}\left(\omega_{s_{-}}^{2}-\omega_{s_{+}}^{2}\right)}\sin\left(\omega_{s_{-}}t\right)+\frac{\omega_{s_{+}}^{2}-\omega_{s}^{2}}{\omega_{s_{+}}\left(\omega_{s_{+}}^{2}-\omega_{s_{-}}^{2}\right)}\sin\left(\omega_{s_{+}}t\right)\right)\frac{1}{R\cos\tilde{\phi }} \end{array}$$
$\delta f_{b,d}$	$$\begin{array}{cc} G_{2,3}\left(s\right) & =0\\ g_{2,3}\left(t\right) & =0 \end{array}$$
$\delta \omega_{ib,n}$	$$\begin{array}{cc} G_{2,4}\left(s\right) & =\frac{\omega_{s}^{2}\left(s^{2}+\omega_{2,4+}^{2}\right)\left(s^{2}+\omega_{2,4-}^{2}\right)}{s\left(s^{2}+\omega_{ie}^{2}\right)\left(s^{2}+\omega_{s_{-}}^{2}\right)\left(s^{2}+\omega_{s_{+}}^{2}\right)}\frac{1}{\cos\tilde{\phi }}\\ g_{2,4}\left(t\right) & =\left(\frac{\omega_{2,4+}^{2}\omega_{2,4-}^{2}}{\omega_{s_{-}}^{2}\omega_{s_{+}}^{2}\omega_{ie}^{2}}-\frac{\omega_{s_{-}}^{4}-\omega_{s_{-}}^{2}\left(\omega_{2,4+}^{2}+\omega_{2,4-}^{2}\right)+\omega_{3}^{2}\omega_{4}^{2}}{\omega_{s_{-}}^{2}\left(\omega_{s_{-}}^{2}-\omega_{s_{+}}^{2}\right)\left(\omega_{s_{-}}^{2}-\omega_{ie}^{2}\right)}\cos\left(\omega_{s_{-}}t\right)\right.\\ \; & \;-\frac{\omega_{s_{+}}^{4}-\omega_{s_{+}}^{2}\left(\omega_{3}^{2}+\omega_{4}^{2}\right)+\omega_{3}^{2}\omega_{4}^{2}}{\omega_{s_{+}}^{2}\left(\omega_{s_{+}}^{2}-\omega_{s_{-}}^{2}\right)\left(\omega_{s_{+}}^{2}-\omega_{ie}^{2}\right)}\cos\left(\omega_{s_{+}}t\right)\\ \; & \left.\;-\frac{\omega_{ie}^{4}-\omega_{ie}^{2}\left(\omega_{3}^{2}+\omega_{4}^{2}\right)+\omega_{3}^{2}\omega_{4}^{2}}{\omega_{ie}^{2}\left(\omega_{ie}^{2}-\omega_{s_{-}}^{2}\right)\left(\omega_{ie}^{2}-\omega_{s_{-}}^{2}\right)}\cos\left(\omega_{ie}t\right)\right)\frac{\omega_{s}^{2}}{\cos\tilde{\phi }}\\ \omega_{2,4\pm }^{2} & =\frac{\omega_{s}^{2}+3\omega_{ie}^{2}\cos^{2}\tilde{\phi }}{2}-\omega_{ie}^{2}\pm \frac{1}{2}\sqrt{{\left(\omega_{ie}^{2}\cos^{2}\tilde{\phi }-2\omega_{ie}^{2}+\omega_{s}^{2}\right)}^{2}-4\omega_{s}^{2}\omega_{ie}^{2}\cos^{2}\tilde{\phi }} \end{array}$$
$\delta \omega_{ib,e}$	$$\begin{array}{cc} G_{2,5}\left(s\right) & =-\frac{3\omega_{s}\omega_{ie}\tan\tilde{\phi }s^{2}}{\left(s^{2}+\omega_{ie}^{2}\right)\left(s^{2}+\omega_{s_{-}}^{2}\right)\left(s^{2}+\omega_{s_{+}}^{2}\right)}-\frac{\omega_{s}^{4}\omega_{ie}\tan\tilde{\phi }}{\left(s^{2}+\omega_{ie}^{2}\right)\left(s^{2}+\omega_{s_{-}}^{2}\right)\left(s^{2}+\omega_{s_{+}}^{2}\right)}\\ g_{2,5}\left(t\right) & =\left(\frac{3\omega_{s_{-}}^{2}-\omega_{s}^{2}}{\omega_{s_{-}}\left(\omega_{s_{-}}^{2}-\omega_{s_{+}}^{2}\right)\left(\omega_{s_{-}}^{2}-\omega_{ie}^{2}\right)}\sin\left(\omega_{s_{-}}t\right)+\frac{3\omega_{s_{+}}^{2}-\omega_{s}^{2}}{\omega_{s_{+}}\left(\omega_{s_{+}}^{2}-\omega_{s_{-}}^{2}\right)\left(\omega_{s_{+}}^{2}-\omega_{ie}^{2}\right)}\sin\left(\omega_{s_{+}}t\right)\right.\\ \; & \;\left.+\frac{3\omega_{ie}^{2}-\omega_{s}^{2}}{\omega_{ie}\left(\omega_{ie}^{2}-\omega_{s_{-}}^{2}\right)\left(\omega_{ie}^{2}-\omega_{s_{+}}^{2}\right)}\sin\left(\omega_{ie}t\right)\right)\omega_{s}^{2}\omega_{ie}\tan\tilde{\phi } \end{array}$$
$\delta \omega_{ib,d}$	$$\begin{array}{cc} G_{2,6}\left(s\right) & =-\frac{3\omega_{s}\omega_{ie}^{2}\sin\tilde{\phi }s}{\left(s^{2}+\omega_{ie}^{2}\right)\left(s^{2}+\omega_{s_{-}}^{2}\right)\left(s^{2}+\omega_{s_{+}}^{2}\right)}-\frac{\omega_{s}^{4}\omega_{ie}^{2}\sin\tilde{\phi }}{\left(s^{2}+\omega_{ie}^{2}\right)\left(s^{2}+\omega_{s_{-}}^{2}\right)\left(s^{2}+\omega_{s_{+}}^{2}\right)}\\ g_{2,6}\left(t\right) & =\left(-\frac{\omega_{s}^{2}}{\omega_{s_{-}}^{2}\omega_{s_{+}}^{2}\omega_{ie}^{2}}+\frac{\omega_{s}^{2}-3\omega_{s_{-}}^{2}}{\omega_{s_{-}}^{2}\left(\omega_{s_{-}}^{2}-\omega_{s_{+}}^{2}\right)\left(\omega_{s_{-}}^{2}-\omega_{ie}^{2}\right)}\cos\left(\omega_{s_{-}}t\right)\right.\\ \; & \;+\frac{\omega_{s}^{2}-3\omega_{s_{+}}^{2}}{\omega_{s_{+}}^{2}\left(\omega_{s_{+}}^{2}-\omega_{s_{-}}^{2}\right)\left(\omega_{s_{+}}^{2}-\omega_{ie}^{2}\right)}\cos\left(\omega_{s_{+}}t\right)\\ \; & \;\left.+\frac{\omega_{s}^{2}-3\omega_{ie}^{2}}{\omega_{ie}^{2}\left(\omega_{ie}^{2}-\omega_{s_{-}}^{2}\right)\left(\omega_{ie}^{2}-\omega_{s_{+}}^{2}\right)}\cos\left(\omega_{ie}t\right)\right)\omega_{s}^{2}\omega_{ie}^{2}\sin\tilde{\phi } \end{array}$$

## Appendix B. Analytical Solution

For impulse responses of the form given in Appendix A, the integral (23) can be solved analytically. For sine-based transfer functions of the general form

(A1) $$g_{a,\sin}\left(t\right)=a_{0}+a_{1}t+a_{2}\sin\left(\omega_{s_{-}}t\right)+a_{3}\sin\left(\omega_{s_{+}}t\right)+a_{4}\sin\left(\omega_{ie}t\right)$$

the integral yields:

(A2) $$\begin{array}{cc} K(t) & =\int_{0}^{t}g_{a,sin}\left(t-\tau \right)g_{b,sin}\left(t-\tau \right)d\tau \\ \; & =\left(a_{0}b_{0}+\frac{a_{2}b_{2}+a_{3}b_{3}+a_{4}b_{4}}{2}\right)t+\left(a_{0}b_{1}+a_{1}b_{0}\right)t^{2}+a_{1}b_{1}t^{3}\\ \; & \;-\frac{a_{2}b_{3}+b_{2}a_{3}}{2(\omega_{s_{-}}+\omega_{s_{+}})}\sin\left((\omega_{s_{-}}+\omega_{s_{+}})t\right)-\frac{a_{2}b_{4}+b_{2}a_{4}}{2(\omega_{s_{-}}+\omega_{ie})}\sin\left((\omega_{s_{-}}+\omega_{ie})t\right)\\ \; & \;-\frac{a_{3}b_{4}+b_{3}a_{4}}{2(\omega_{s_{+}}+\omega_{ie})}\sin\left((\omega_{s_{+}}+\omega_{ie})t\right)+\frac{a_{2}b_{3}+b_{2}a_{3}}{2(\omega_{s_{-}}-\omega_{s_{+}})}\sin\left((\omega_{s_{-}}-\omega_{s_{+}})t\right)\\ \; & \;+\frac{a_{2}b_{4}+b_{2}a_{4}}{2(\omega_{s_{-}}-\omega_{ie})}\sin\left((\omega_{s_{-}}-\omega_{ie})t\right)+\frac{a_{3}b_{4}+b_{3}a_{4}}{2(\omega_{s_{+}}-\omega_{ie})}\sin\left((\omega_{s_{+}}-\omega_{ie})t\right)\\ \; & \;-\frac{a_{2}b_{2}}{4\omega_{s_{-}}}\sin\left(2\omega_{s_{-}}t\right)-\frac{a_{3}b_{3}}{4\omega_{s_{+}}}\sin\left(2\omega_{s_{+}}t\right)-\frac{a_{4}b_{4}}{4\omega_{ie}}\sin\left(2\omega_{ie}t\right)\\ \; & \;+2\frac{a_{0}b_{2}+b_{0}a_{2}+\left(a_{1}b_{2}+b_{1}a_{2}\right)t}{\omega_{s_{-}}}\sin{\left(\frac{\omega_{s_{-}}}{2}t\right)}^{2}\\ \; & \;+2\frac{a_{0}b_{3}+b_{3}a_{0}+\left(a_{1}b_{3}+b_{1}a_{3}\right)t}{\omega_{s_{+}}}\sin{\left(\frac{\omega_{s_{+}}}{2}t\right)}^{2}\\ \; & \;+2\frac{a_{0}b_{4}+b_{4}a_{0}+\left(a_{1}b_{4}+b_{1}a_{4}\right)t}{\omega_{ie}}\sin{\left(\frac{\omega_{ie}}{2}t\right)}^{2} \end{array}$$

In a similar fashion, the integral over a cosine-based transfer function

(A3) $$g_{a,\cos}\left(t\right)=a_{0}+a_{1}t+a_{2}\cos\left(\omega_{s_{-}}t\right)+a_{3}\cos\left(\omega_{s_{+}}t\right)+a_{4}\cos\left(\omega_{ie}t\right)$$

is given as:

(A4) $$\begin{array}{cc} K(t) & =\int_{0}^{t}g_{a,Cos}\left(t-\tau \right)g_{b,Cos}\left(t-\tau \right)d\tau \\ \; & =\left(a_{0}b_{0}+\frac{a_{2}b_{2}+a_{3}b_{3}+a_{4}b_{4}}{2}\right)t+\left(a_{0}b_{1}+a_{1}b_{0}\right)t^{2}+a_{1}b_{1}t^{3}\\ \; & \;+\frac{a_{2}b_{3}+b_{2}a_{3}}{2(\omega_{s_{-}}+\omega_{s_{+}})}\sin\left((\omega_{s_{-}}+\omega_{s_{+}})t\right)+\frac{a_{2}b_{4}+b_{2}a_{4}}{2(\omega_{s_{-}}+\omega_{ie})}\sin\left((\omega_{s_{-}}+\omega_{ie})t\right)\\ \; & \;+\frac{a_{3}b_{4}+b_{3}a_{4}}{2(\omega_{s_{+}}+\omega_{ie})}\sin\left((\omega_{s_{+}}+\omega_{ie})t\right)+\frac{a_{2}b_{3}+b_{2}a_{3}}{2(\omega_{s_{-}}-\omega_{s_{+}})}\sin\left((\omega_{s_{-}}-\omega_{s_{+}})t\right)\\ \; & \;+\frac{a_{2}b_{4}+b_{2}a_{4}}{2(\omega_{s_{-}}-\omega_{ie})}\sin\left((\omega_{s_{-}}-\omega_{ie})t\right)+\frac{a_{3}b_{4}+b_{3}a_{4}}{2(\omega_{s_{+}}-\omega_{ie})}\sin\left((\omega_{s_{+}}-\omega_{ie})t\right)\\ \; & \;+\frac{a_{2}b_{2}}{4\omega_{s_{-}}}\sin\left(2\omega_{s_{-}}t\right)+\frac{a_{3}b_{3}}{4\omega_{s_{+}}}\sin\left(2\omega_{s_{+}}t\right)+\frac{a_{4}b_{4}}{4\omega_{ie}}\sin\left(2\omega_{ie}t\right)\\ \; & \;+\frac{a_{0}b_{2}+b_{0}a_{2}+\left(a_{1}b_{2}+b_{1}a_{2}\right)t}{\omega_{s_{-}}}\sin\left(\omega_{s_{-}}t\right)\\ \; & \;+\frac{a_{0}b_{3}+b_{3}a_{0}+\left(a_{1}b_{3}+b_{1}a_{3}\right)t}{\omega_{s_{+}}}\sin\left(\omega_{s_{+}}t\right)\\ \; & \;+\frac{a_{0}b_{4}+b_{4}a_{0}+\left(a_{1}b_{4}+b_{1}a_{4}\right)t}{\omega_{ie}}\sin\left(\omega_{ie}t\right) \end{array}$$

## Appendix C. IEEE Sensor Noise Processes

**Table A3:** Power spectral density and Allan variance of typical inertial sensor noise processes [20].

Process	Power Spectral Density	Allan Variance
Angular Random Walk	$S_{N}f=N^{2}$	$\sigma_{N}^{2}\tau =\frac{N^{2}}{\tau }$
*White noise*		
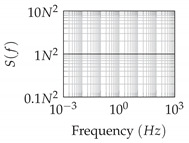
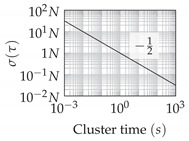
Rate Random Walk	$S_{K}f=\frac{K^{2}}{{\left(2\pi f\right)}^{2}}$	$\sigma_{K}^{2}\tau =\frac{K^{2}\tau }{3}$
*Brownian noise*		
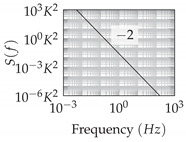
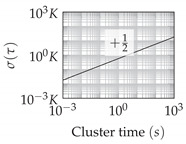
Rate Ramp Noise	$S_{R}\left(f\right)=\frac{R^{2}}{{\left(2\pi f\right)}^{3}}$	$\sigma_{K}^{2}\left(\tau \right)=\frac{R^{2}\tau^{2}}{2}$

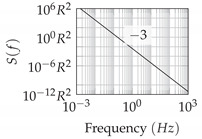
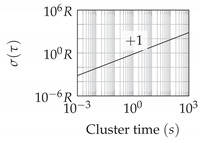
		
Bias Instability	$S_{B}\left(f\right)=\left\{\begin{array}{cc} \left(\frac{B^{2}}{2\pi }\right)\frac{1}{f} & f\leq f_{0,B}\\ 0 & f>f_{0,B} \end{array}\right.$	$\sigma_{B}^{2}\left(f\right)=\frac{2B^{2}}{\pi }\left[\ln2-\frac{\sin^{3}\left(x\right)}{2x^{2}}\left(\sin(x)+4x\cos(x)\right)\right.$
		$\left.+Ci(2x)-Ci(4x)\right],\;x=\pi f_{0,B}\tau$
*Band limited pink noise, flicker noise*		
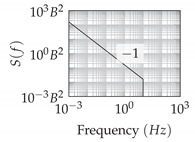
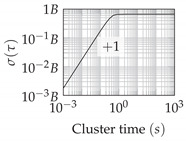
Quantization Noise	$S_{Q}\left(f\right)\approx Q^{2}\tau_{s}{\left(2\pi f\right)}^{2}$	$\sigma_{Q}^{2}\left(\tau \right)=\frac{3Q^{2}}{\tau^{2}}$
*Violet noise*		
